# MATLAB Simulation of UPQC for Power Quality Mitigation Using an Ant Colony Based Fuzzy Control Technique

**DOI:** 10.1155/2015/304165

**Published:** 2015-10-01

**Authors:** N. Kumarasabapathy, P. S. Manoharan

**Affiliations:** ^1^University VOC College of Engineering, Thoothukudi 628008, India; ^2^Thiagarajar College of Engineering, Madurai 625015, India

## Abstract

This paper proposes a fuzzy logic based new control scheme for the Unified Power Quality Conditioner (UPQC) for minimizing the voltage sag and total harmonic distortion in the distribution system consequently to improve the power quality. UPQC is a recent power electronic module which guarantees better power quality mitigation as it has both series-active and shunt-active power filters (APFs). The fuzzy logic controller has recently attracted a great deal of attention and possesses conceptually the quality of the simplicity by tackling complex systems with vagueness and ambiguity. In this research, the fuzzy logic controller is utilized for the generation of reference signal controlling the UPQC. To enable this, a systematic approach for creating the fuzzy membership functions is carried out by using an ant colony optimization technique for optimal fuzzy logic control. An exhaustive simulation study using the MATLAB/Simulink is carried out to investigate and demonstrate the performance of the proposed fuzzy logic controller and the simulation results are compared with the PI controller in terms of its performance in improving the power quality by minimizing the voltage sag and total harmonic distortion.

## 1. Introduction

Power quality is one of the major concerns in the present era. It has become important, especially with the introduction of sophisticated devices, whose performance is very sensitive to the quality of power supplied. Power quality problem is an occurrence manifested as a nonstandard voltage, current, or frequency that results in a failure of end use equipment [1]. The main objective of devising UPQC is the combined use of series-active and shunt-active filters chiefly to compensate negative-sequence current and harmonics as the SCR controlled capacitor banks compensate reactive power in power frequency [2]. Modeling and simulation of custom power conditioners happen to be inevitable as power electronics-based equipment in use for augmenting power quality in distribution networks [3]. Extended merit of the UPQC includes the following: it has a similar feature to SCR controlled capacitor banks of achieving load compensation resulting in drawing the balanced sinusoidal currents in the current control mode [4, 5]. In addition it is also the most comprehensive power quality enhancing device for susceptible nonlinear loads, which need exact sinusoidal input supply [6].

UPQC can also be operated in different possible configurations for single-phase (2-wire) and 3-phase (3-wire and 4-wire) networks, diverse compensation approaches, and recent developments are also found in the field [7]. Thus this conditioner can achieve reasonable power quality improvement, reducing the power disturbances that are supplied to the customers by the mains using the series unit. Additional facilities for PQ (i.e., mains power interruptions) can be offered to the customers (custom power) from the shunt units [8]. To reiterate again the main principle of UPQC is to ensure quality supply voltage and load current disturbances, namely, sags, swell, imbalance, harmonics, reactive currents, and current unbalance produced by the nonlinear loads [9, 10].

The control signal given to the UPQC plays a significant role in establishing a better operation of the device. Conventional control schemes are widely used in addition to the control schemes derived from artificial intelligence which is also recorded in the literature. Applications of some advanced mathematical tools in general, and wavelet transform in particular, in power quality are also applied. An extensive collection of literature covering applications of fuzzy logic, expert systems, neural networks, and genetic algorithms in power quality is also growing [11]. The ANN-based controller is designed for the current control of the shunt-active power filter and trained offline using data from the conventional proportional-integral controller [12]. Similarly, a fully digital controller based on the TMS320F2812 DSP platform which is implemented for the reference generation as well as control purposes is proposed [13].

In another attempt [14], the authors propose the application of fuzzy logic (FL) technique within microgrid energy system based on the most modern power conditioning equipment devices such as Unified Power Quality Conditioner (UPQC). Similarly [15, 16], a linear quadratic regulator (LQR) control technique embedded with the ANN is used to coordinate the operation of the series and shunt VSIs of the UPQC. In another development a control algorithm based on wavelet transform for UPQC to suppress current harmonics and voltage sags is proposed [17]. Subsequently in [18], the authors emphasize enhancement of power quality by using UPQC with fuzzy logic controller (FLC) and artificial neural network (ANN) controller on the conventional proportional-integral (PI) controller. In [19], the determination of voltage references for series-active power filter is done based on a robust three-phase digital phase locked loop (PLL) system using fuzzy regulator.

In [20], a particle swarm optimization (PSO) method is used to find the solution of the objective function derived for minimizing real power injection of UPQC along with the constraints. Adaptive neurofuzzy inference systems have been used to make the proposed methodology online for minimum real power injection with UPQC by using the PSO-based data for different voltage sag conditions. A fuzzy logic with PWM technique for UPQC for power quality improvement is discussed in [21]. In another extension [22], a fuzzy-expert system for automated detection and classification of power quality disturbances is presented. The paper [23] focuses on the application of wavelet transform technique to extract features from power quality disturbance waveforms and their classification using a combination of artificial neural network and fuzzy logic. Simulations are carried out with PI as well as fuzzy logic controller for both *p*-*q* and *Id*-*Iq* control strategies by considering different voltage conditions and adequate results were presented in [24]. Thus the main focus of this proposed system is to improve the power quality by compensating the voltage sag and eliminating the harmonics in the distribution network using a fuzzy logic [25, 26] based technique. In this research the fuzzy membership functions are carried out by using an ant colony optimization [27] technique for optimal fuzzy logic control.

The simulation is done using MATLAB and Simulink Toolbox for both control schemes used for the generation of control signals for the UPQC. The rest of the paper is organized in such a way that UPQC is revisited in Section 2. Control strategy and necessary equations of the UPQC are discussed in Section 3. Fuzzy logic and its features used in this research are presented in Section 4. The proposed FL-ACO method is discussed in Section 5. Numerical simulations are shown in Section 6 and the discussions and conclusions follow finally.

## 2. Unified Power Quality Conditioner: An Overview

UPQC is one of the custom power devices used at the electrical power distribution systems to improve the power quality of distribution system customers [2]. UPQC could be used to cancel current harmonics, to compensate reactive power, to eliminate voltage harmonics, to improve voltage regulation, to correct voltage and current imbalances, to correct voltage sag or swell, and to avoid voltage interruptions [3]. UPQC consists of both shunt and series compensators. A shunt compensator is used to cancel the disturbances in current whereas series compensator is used to cancel disturbances in voltage. Shunt compensator could be connected to the left or right of the series compensator. Ideally, shunt compensator injects current to achieve purely balanced sinusoidal source currents in phase with the supply voltages at rated magnitude and frequency. On the other hand, series compensation is used to inject voltage to maintain terminal voltage at rated magnitude and frequency.

**Figure 1:**
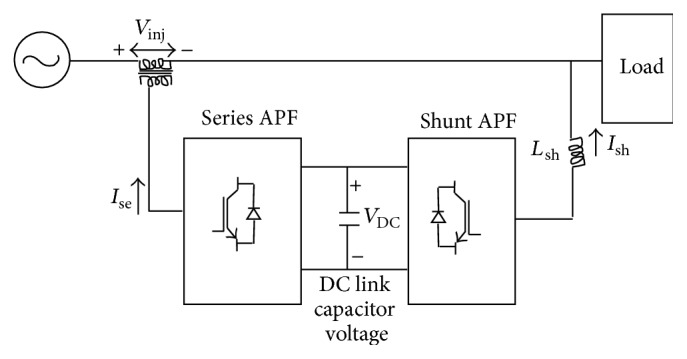
Schematic diagram of the UPQC.

The schematic diagram of a three-phase UPQC is shown in Figure 1. Voltage source inverters are used for shunt and series compensation. One may note that both voltage source inverters are supplied from a common DC link capacitor. One of the voltage source inverters is connected in parallel with the AC system while the other one is connected in series with the AC system through injection transformers. The inverter connected in parallel, together with its control circuit, forms the shunt compensation circuit. On the other hand, the inverter connected in series with appropriate control circuit forms the series compensation circuit. For the successful operation of the UPQC, the DC capacitor voltage should be at least 150% of the maximum line-line supply voltage. To regulate the capacitor voltage constant, either a PI controller or a fuzzy logic controller could be used. Thus in this research a fuzzy logic integrated with ACO is proposed for the control of UPQC. The expression is given by

(1) $$\begin{array}{c} V_{\text{inj}}^{2}=V_{\text{op}}^{2}x^{2}+2V_{\text{op}}^{2}\left(\;1-x\;\right)\left(\;1-\cos\beta \;\right), \end{array}$$

where *V*_op_ is the output required voltage and *β* is the angle of voltage. Hence,

(2) VA−UPQC=VopIopG1θ,x,β+G2θ,β+Iop2G22θ,βZSLC.

This is the final apparent power expression for the UPQC.

## 3. Control Strategy of UPQC

**Figure 2:**
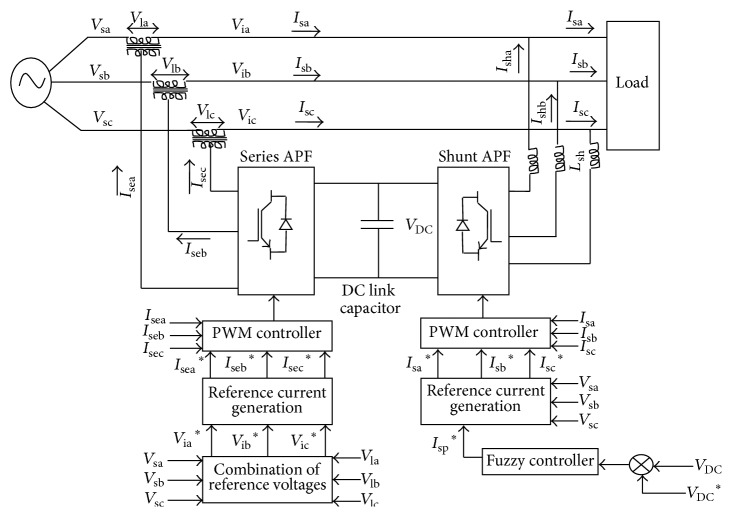
Control scheme of UPQC.

The control scheme of three-phase UPQC is shown in Figure 2. It consists of shunt APF and series APF. Both the shunt and series APFs are current controlled. The shunt APF is indirect current controlled.

### 3.1. Fundamental Phasor Configuration

The fundamental description of the UPQC with an ideal voltage source and the fuzzy logic is modeled as an ideal current source. The UPQC source voltage (*V*_*S*_) is taken as the reference and is given by [28]

(3) $$\begin{array}{c} V_{\text{in}}∠\theta =V_{\text{pen}}∠\beta +V_{\text{ph}}∠\delta , \end{array}$$

where *V*_ph_ is the load voltage and *V*_pen_ is the penetrated voltage of the UPQC. Note that for a power factor given load and voltage sag requirement to determine the magnitude and constant phase angles of *V*_pen_ to maintain the load voltage constant at its rated valued. There are an infinite number of solutions for *V*_pen_. The novel control strategy proposed in this paper precalculated this angle of injection *γ* to optimize the loading of the UPQC. The load current is given by

(4) $$\begin{array}{c} I_{\text{ph}}∠\delta -\theta =I_{\text{in}}∠\theta +I_{C}∠\delta . \end{array}$$

The angle *ϕ* represents the lagging angle of the load current (*I*_ph_) with respect to *V*_ph_. As assumed power supply only active component of the load current, *δ* = 0. The current *I*_*C*_ is preferably the reactive load current component. The operation of the UPQC by means of a phasor diagram is easy to understand. The suffix “ph” denotes the load terms and “in” denotes the supply terms. Suffixes 1 and 2 denote two stages in time, on the other state of the power supply for UPQC taking action. Initially in Stage 1, the supply voltage has no deficiency:

(5) $$\begin{array}{c} V_{\text{in}}=V_{\text{ph}1}=V_{\text{in}1}=\text{Constant}=V_{0}. \end{array}$$

Corresponding to that situation, the SLCVC current is given by *I*_*C*1_, where angle *β* = +90; in advance of the supply voltage, it is assumed that the power factor is lagging.

**Figure 3:**
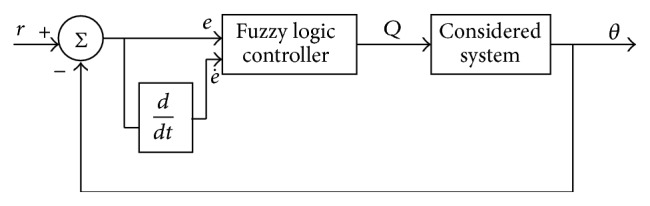
Fuzzy logic controller for system under consideration.

In Stage 2 the supply voltage magnitude has reduced to *V*_in2_, which requires the UPQC to take action such that *V*_ph2_ is restored to its original magnitude (|*V*_ph1_| = |*V*_ph2_|). This is achieved by injecting the series voltage *V*_inj_ *∠γ*, and load voltage can be obtained by selecting *γ* in a range from 0 to 90°, together with the appropriate magnitude of *V*_inj_. In Stage 2, according to Figure 3,

(6) Iin2cos⁡β=Iph2cos⁡θ.

(7) IC2=Iph2sin⁡θ−βcos⁡β.

If *α* < *ϕ*, the two active filters inject the load reactive power. Interestingly, this sagging voltage is such that, for a load power factor given, the angle *β* becomes equal to *θ*, and then from (6), it can be inferred that *I*_*S*2_ = *I*_*L*2_. The load current is understood to be constant:

(8) $$\begin{array}{c} I_{L1}=I_{L2}=I_{0} \end{array}$$

with a fundamental lagging power factor of cos⁡*ϕ*. The active demand for power in the load is constant and equal to what to do with the source:

(9) $$\begin{array}{c} V_{S}I_{S}=V_{L}I_{L}\cos\phi =\text{Constant}. \end{array}$$

In the case of sag, where |*V*_*S*2_| < |*V*_*S*1_| and *x* is the per unit voltage sag,

(10) $$\begin{array}{c} V_{S2}=\left(\;1-x\;\right)V_{S1}=\left(\;1-x\;\right)V_{0}. \end{array}$$

To maintain a constant active power in a voltage sag, *V*_*S*1_ *I*_*S*1_ = *V*_*L*2_ *I*_*L*2_ and

(11) $$\begin{array}{c} I_{S2}=\frac{V_{0}I_{L}\cos\phi }{V_{0}\left(1-x\right)}=I_{0}\frac{\cos\phi }{\left(1-x\right)}. \end{array}$$

Thus,

(12) VL2sin⁡α=Vinjsin⁡γ

(13) VL2cos⁡α=VS2+Vinjcos⁡γ

or

(14) sin⁡γ=VL2Vinjsin⁡α;cos⁡γ=Vinj2−VL22sin2⁡αVinj.

From (7) and (12),

(15) $$\begin{array}{c} V_{\text{inj}}^{2}=V_{0}^{2}x^{2}+2V_{0}^{2}\left(\;1-x\;\right)\left(\;1-\cos\alpha \;\right). \end{array}$$

The apparent power of the series compensator is expressed by

(16) $$\begin{array}{c} \left|\;V_{\text{inj}}\;\right|\left|\;I_{S2}\;\right|=V_{0}I_{0}\cos\phi \frac{\sqrt{x^{2}+2\left(1-x\right)\left(1-\cos\alpha \right)}}{\left(1-x\right)} \end{array}$$

which yields

(17) $$\begin{array}{c} I_{C2}\cos\alpha =I_{L2}+V_{\text{inj}}\sin\left(\;\phi -\alpha \;\right). \end{array}$$

Therefore, the apparent power rating is modeled by

(18) IC2VL2+IC22ZSLC=IL2V0sin⁡ϕ−αcos⁡α+IL2sin⁡ϕ−α2cos⁡αZSLC,

where *Z*_SLC_ is impedance of the synchronous link converter in p.u. summing up (16) and (18); the total apparent power rating of the UPQC is expressed as

(19) VA−UPQC=VopIopG1θ,x,β+G2θ,β+Iop2G22θ,βZSLCG1θ,x,β=cos⁡θx2+21−x1−cos⁡β1−xG2θ,β=sin⁡ϕ−βcos⁡β.

Therefore, for a given load power factor angle *ϕ* and voltage sag *x* p.u., the VA loading will be a function of the advance angle *α* of *V*_*L*_. The minimum VA occur when *ϕ* = *α*, so that *I*_*S*2_ = *I*_*L*2_ and *I*_*C*2_ = 0. Under these conditions, the optimal injected voltage is given by

(20) $$\begin{array}{c} V_{\text{inj}}=V_{0}\sqrt{x^{2}+2\left(1-x\right)\left(1-\cos\phi \right)}. \end{array}$$

And the voltage advance angle for the SC is given by

(21) $$\begin{array}{c} \sin\gamma =\frac{\sqrt{\left(1-\cos^{2}\alpha \right)}}{\sqrt{x^{2}+2\left(1-x\right)\left(1-\cos\phi \right)}}. \end{array}$$

Therefore, (20) and (21) are the control equations which will decide that the optimum angle of voltage injection by the UPQC could be obtained through the proposed ACO-FL control technique (Figure 12).

## 4. Fuzzy Logic Controller

In FLC, basic control action is determined by a set of linguistic rules. These rules are determined by the system. Since the numerical variables are converted into linguistic variables, mathematical modelling of the system is not required in FLC.

The FLC comprises three parts: fuzzification, interference engine, and defuzzification.

### 4.1. Fuzzification

Membership function values are assigned to the linguistic variables, using seven fuzzy subsets: NB (Negative Big), NM (Negative Medium), NS (Negative Small), ZE (Zero), PS (Positive Small), PM (Positive Medium), and PB (Positive Big). The partition of fuzzy subsets and the shape of membership function adapt the shape up to appropriate system. The values of input error *E*(*k*) and change in error CE(*k*) are normalized by an input scaling factor shown in Figure 3.

In this system the input scaling factor has been designed such that input values are between −1 and +1. The triangular shape of the membership function of this arrangement presumes that for any particular input there is only one dominant fuzzy subset. The input error *E*(*k*) for the FLC can be calculated from the maximum power point as given in (20) and (21):

(22) Ek=Pphk−Pphk−1Vphk−Vphk−1CEk=Ek−Ek−1.

### 4.2. Interference Method

Several composition methods such as Max-Min and Max-Dot have been proposed in the literature. In this paper Max-Min method is used. The output membership function of each rule is given by the minimum operator and maximum operator.  Table 1 shows rule base of the FLC.

### 4.3. Defuzzification

As a plant usually requires a nonfuzzy value of control, a defuzzification stage is needed. To compute the output of the FLC, height method is used and the FLC output modifies the control output. Further, the output of FLC controls the switch in the inverter.

**Figure 4:**
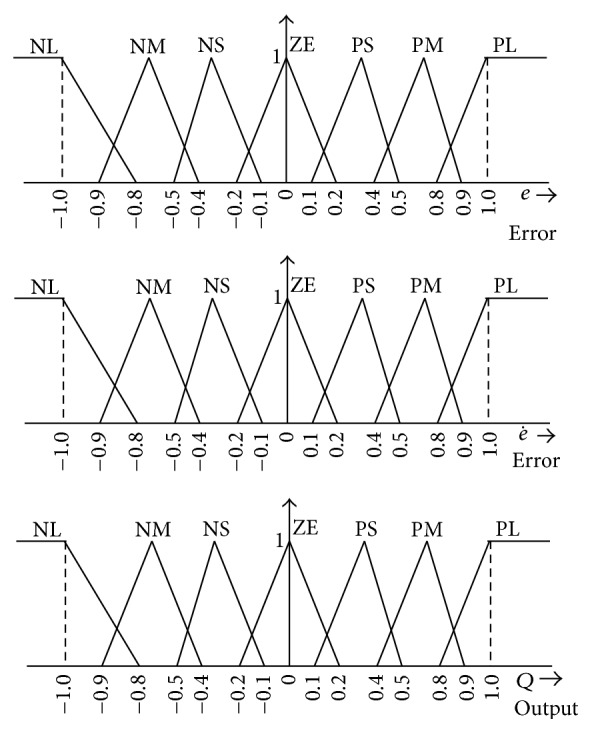
Membership functions of *e*, $\dot{e}$, and *Q*.

The FLC uses a rule base as shown in Table 1 and the membership function as shown in Figure 4. The FLC is characterized as (i) seven fuzzy sets for each input and output, (ii) triangular membership functions for simplicity, (iii) fuzzification using continuous universe of discourse, (iv) implication using Mamdani's “min” operator, and (v) defuzzification using the “height” method. In UPQC, the active power, reactive power, terminal voltage of the line, and capacitor voltage are required to be maintained. In order to control these parameters, they are sensed and compared with the reference values. To achieve this, the membership functions of FLC are error, change in error, and output. In the present work, for fuzzification, nonuniform fuzzifier has been used. If the exact values of error and change in error are small, they are divided conversely and if the values are large, they are divided coarsely. The set of FLC rules is derived from

(23) $$\begin{array}{c} u=-\left[\;\alpha E+\left(\;1-\alpha \;\right)C\;\right], \end{array}$$

where *α* is called the self-adjustable factor which can regulate whole region of operation, *E* is the error of the system, *C* is the varying ratio error, and *u* is the control variable. A large value of error *E* indicates that the given system is not in the balanced state. If the system is unbalanced, the controller should enlarge its control variables to balance the system as early as possible. One the other hand, small value of the error *E* indicates that the system is near to balanced state. Overshoot plays an important role in the system stability. Less overshoot is required for system stability and in restraining oscillations. In such conditions, *C* in (11) plays an important role, while the role of *E* is diminished. The optimization is done by *α*. During the process, it is assumed that the UPQC neither absorbs active power nor supplies active power during normal conditions. So the active power flowing through the UPQC is assumed to be constant. The control surface of the proposed FLC is shown in Figure 4. It indicates two inputs, one output, and a surface showing input-output mapping. The proposed defuzzification method is briefed in the next section.

### 4.4. Weighted Average Method

This is a method that cannot be used for asymmetrical output membership functions and can be used only for symmetrical output membership functions. Weighting each membership function in the obtained output by its largest membership value forms this method. The evaluation expression for this method is

(24) $$\begin{array}{c} z^{*}=\frac{\sum \mu_{\underset{\sim }{c}}\left(\bar{z}\right)\bar{z}}{\sum \mu_{\underset{\sim }{c}}\left(\bar{z}\right)}, \end{array}$$

where ∑ is used for algebraic sum.

**Figure 5:**
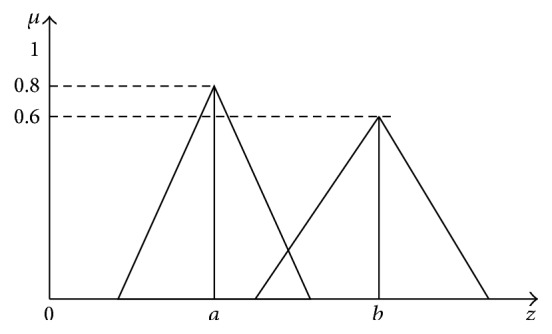
Weighted average method.

From Figure 5, the defuzzified value can be calculated using (23) as follows:

(25) $$\begin{array}{c} z^{*}=\frac{a\left(0.8\right)+b\left(0.65\right)}{0.8+0.65}. \end{array}$$

## 5. Ant Colony Optimization Algorithm

Ant Colony Optimization (ACO) algorithm is essentially a system based on agents which simulate the natural behavior of ants, including mechanisms of cooperation and adaptation. It is designed to reproduce the ability of ant colonies to determine the shortest paths to food. Real ants can indirectly communicate by pheromone information without using visual cues and are capable of finding the shortest path between food sources and their nests. The ant deposits pheromone on the trail while walking, and the other ants follow the pheromone trails with some probability which are proportioned to the density of the pheromone. Through this mechanism, ants will eventually find the shortest path. Artificial ants imitate the behavior of real ants of how they forage the food but can solve much more complicated problems than real ants can.

ACO algorithms are based on the following ideas:

- Each path followed by an ant is associated with a candidate solution for a given problem.
- When an ant follows a path, the amount of pheromone deposited on that path is proportional to the quality of the corresponding candidate solution for the target problem.
- When an ant has to choose between two or more paths, the path(s) with a larger amount of pheromone have a greater probability of being chosen by the ant.

This algorithm is most simple and involves only a few steps for finding challenging solutions even in complex domain. ACO is used to find the fuzzy membership functions for the control of UPQC.

### 5.1. The Proposed Flow Diagram for the ACO-FL Control Technique

**Figure 6:**
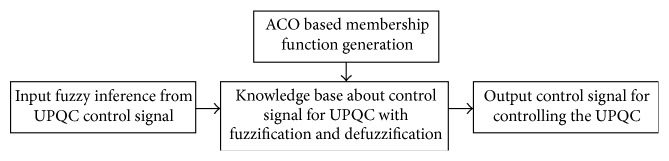
The proposed flow diagram for the ACO-FL control technique.

Figure 6 is implemented in MATLAB/Simulink and the results are summarized in the next section.

## 6. Simulation and Results

**Figure 7:**
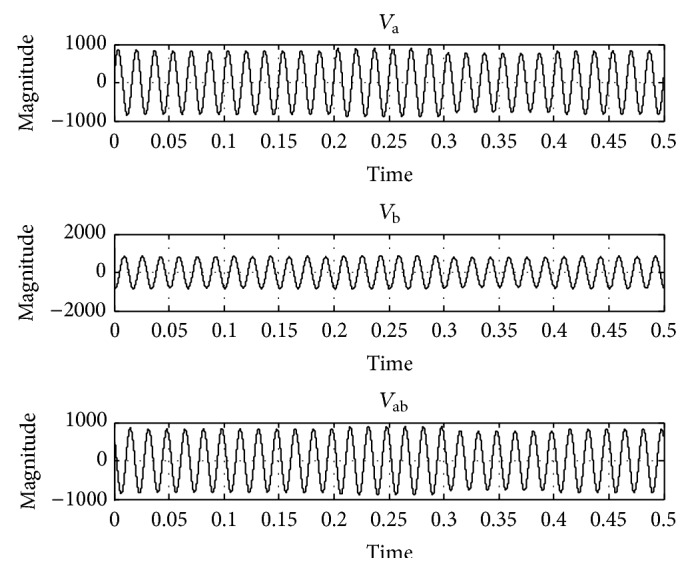
Output waveform without UPQC connected to the system.

**Figure 8:**
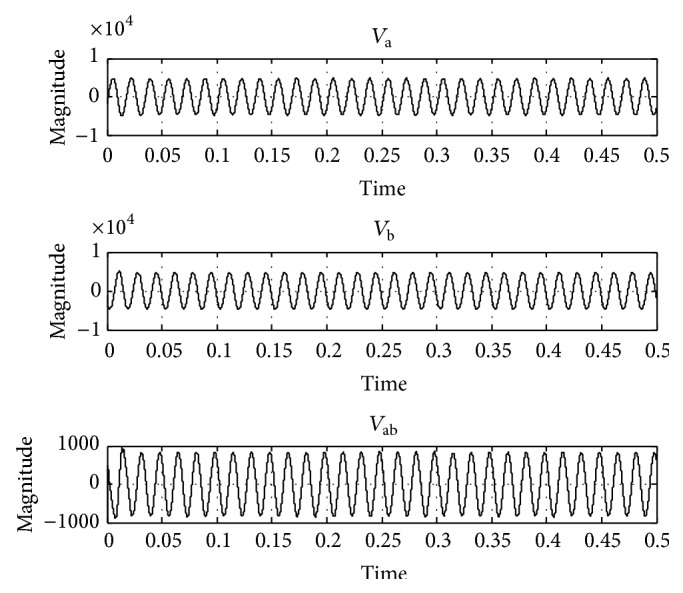
Output waveform with UPQC connected to the system controlled by PI controller.

**Figure 9:**
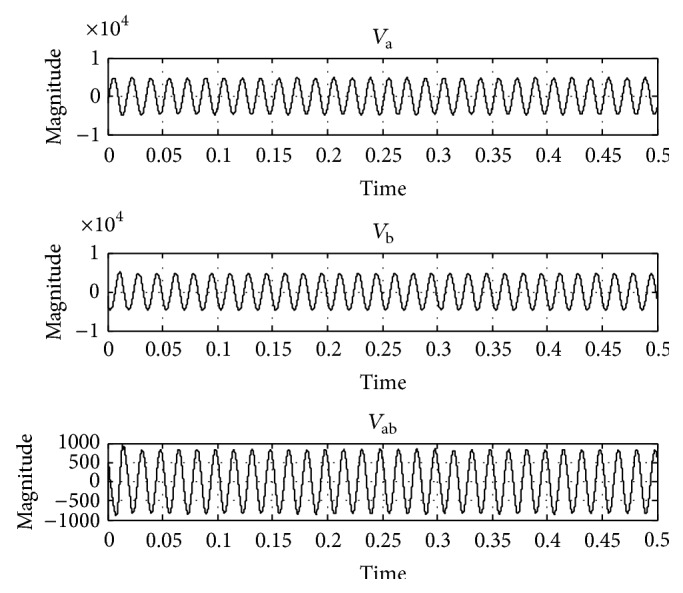
Output waveform with UPQC connected controlled by ACO-FLC technique.

**Figure 10:**
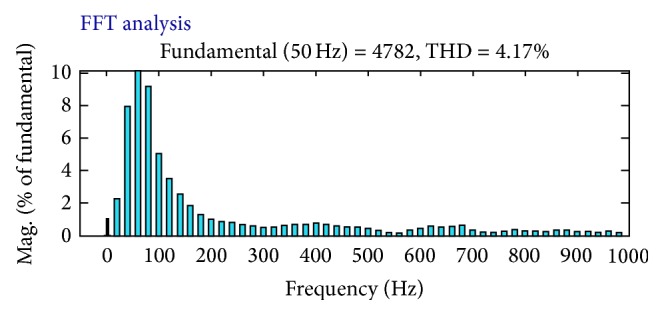
THD when UPQC is not connected to the system.

**Figure 11:**
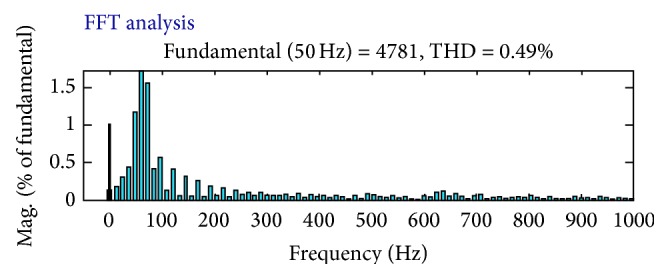
THD when UPQC is tuned by PI.

**Figure 12:**
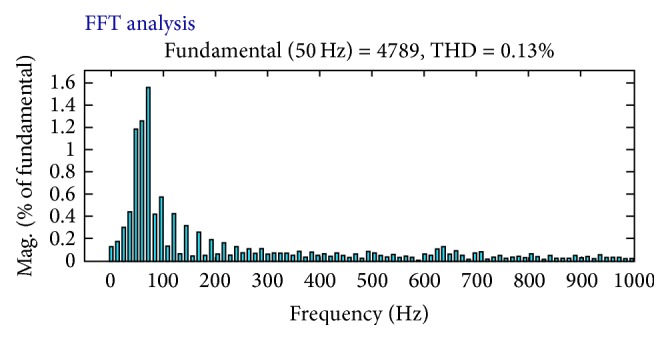
THD when UPQC is tuned by ACO-FLC technique.

In order to test the performance of the UPQC using the proposed FLC, it has been simulated for a 400 V, 50 Hz three-phase AC supply using MATLAB/Simulink. A three-phase diode rectifier feeding an RLC load is considered as nonlinear load. The maximum load power demand is considered as 11 kW + j12 kVAR. The value of source resistance *R*_*S*_ = 0.2 Ω and value of source inductance *L*_*S*_ = 0.2 mH. DC link capacitor value is 10 mF. To test the operation of UPQC under the voltage sag condition, 20% sag in line voltage has been created and simulated, the corresponding voltage waveforms *V*_a_, *V*_b_, and *V*_ab_ are taken and shown in Figure 6, and the THD in the system is 4.17% as shown in Figure 7. Again under 20% voltage sag condition the performance of UPQC has been simulated using the PI controller (Figure 8) and its corresponding output voltage waveforms *V*_a_, *V*_b_, and *V*_ab_ are taken and shown in Figure 9; the voltage sag has been compensated and THD have reduced to 0.49% as shown in Figure 10. When the proposed FLC incorporated UPQC is connected to the system at 0.1 s the simulations are carried out; its corresponding output voltage waveforms *V*_a_, *V*_b_, and *V*_ab_ are taken and shown in Figure 10; the voltage sag has been compensated and THD have reduced to 0.13% as shown in Figure 11. The DC link capacitor voltage is held constant at its reference value by the FLC.

### 6.1. Discussion

By observing Figure 6, it can be noted that the voltage at *V*_b_ is sagging without the UPQC being connected. This situation will be very serious in a power system and leads to imbalance in the system and may cause severe power quality issues in the overall distribution system. To justify the robustness of the control algorithm for voltage harmonic mitigation, a three-phase SCR rectifier with resistive load on the DC side is connected. Thus, the distortion voltage across the load is introduced. To envisage the performance of the shunt APF and series APF independently, both APFs are made to work at different time intervals. Initially the shunt APF is connected for operation. As shown in Figure 6, the supply currents are normal, sinusoidal, and phase line, with the voltages even under unbalanced system voltage condition. The source current THD in phase “c” is enhanced from 12.28% to 4.15%. Later the series APF is made to operate for conditioning. The series-active power filter starts compensating for the voltage harmonics by penetrating instantaneously the phase harmonic voltage, confirming that the load voltage gets no distortion.

To substantiate this in Figure 9, a small distortion in the DC voltages is introduced, but the DC link is able to regulate the DC voltage only to its past magnitude. Figure 10 conveys that a flow of current in the neutral conductor is circulating which has to be suppressed by the neutral phase of the shunt APF, thus forcing the supply-neutral current to zero.  Table 2 summarizes the voltage sag mitigation of various methods.

Due to the operation of the UPQC, the shunt AF current (ica) compensates the reactive power and the harmonics of the load; thus the source current follows the sinusoidal reference currents. The THD of the supply current is 3.19%, while the load current THD is 12.73% as shown in Figures 10 and 11, respectively. Moreover, the peak value of the supply current after compensation is less than the peak value of the load current, thereby increasing the loading capability of the AC mains. The RMS value of phase “a” supply current after compensation is reduced to 27.62 A and before compensation its value is 39.52 A as observed from simulation results. The UPQC effectively maintains the sinusoidal supply currents and a constant DC bus voltage when the load is reduced at 0.52 sec and increased at 0.8 sec as shown in Figure 10. Due to the connection of a single-phase diode rectifier, a current of 17.9 A flows in the load neutral conductor. This current is compensated for by the fourth leg of the shunt AF, thus reducing the supply-neutral current to zero.  Table 3 shows the comparison of THD produced by various methods.

## 7. Conclusion

The confrontation in improving the power quality has become a promising area of research amongst power system and power electronic engineers and researchers. With the ever increasing advent of nonlinear loads and also due to high frequency switching characteristics, suitable conditioners are always a demand. Unified Power Quality Conditioner (UPQC) is one of the promising power electronic circuit modules to overcome voltage sag and total harmonic distortion problems, as the circuit is modeled using both series-active and shunt-active power filters. Thus the benefits of both the power filters are integrated for better power quality mitigation is realized. This paper considers the advantages of the fuzzy logic and proposes a new control scheme for the Unified Power Quality Conditioner (UPQC) for minimizing the voltage sag and total harmonic distortion in the distribution system. The reference signal generated by the fuzzy logic controller was given as input to the UPQC switching module. Exhaustive simulation experiments using MATLAB/Simulink have been done and based on the results and output received it is observed that the proposed fuzzy logic controller is better in improving the power quality by minimizing the voltage sag and total harmonic distortion when compared to the conventional PI controller. To enable this, a systematic approach for creating the fuzzy membership functions is carried out by using an ant colony optimization technique for optimal fuzzy logic control. In this research the UPQC is only simulated for minimizing the voltage sag and total harmonic distortion in the distribution system but the main purpose of UPQC is to compensate for voltage imbalance, reactive power, negative-sequence current, and harmonics and these parameters will be considered in the near future.

## Figures and Tables

**Table 1:** Fuzzy rules base.

*e*	$\dot{e}$						
	NL	NM	NS	ZE	PS	PM	PL
NL	NL	NL	NL	NM	NS	NS	ZE
NM	NL	NM	NM	NM	NS	ZE	ZE
NS	NM	NM	NS	NS	ZE	ZE	PS
ZE	NS	NS	ZE	ZE	ZE	PS	PS
PS	NS	ZE	ZE	PS	PS	PM	PM
PM	ZE	ZE	PS	PM	PM	PM	PL
PL	ZE	PS	PM	PM	PL	PL	PL

**Table 2:** Voltage sag mitigation performance.

System without UPQC	UPQC with PI controller	System with ACO-FLC controller
Voltage sag persists	Voltage sag mitigated	Voltage sag mitigated with high accuracy

**Table 3:** Total harmonic distortion for the proposed system.

THD without UPQC	THD with PI controller	THD with ACO-FLC controller
4.17%	0.49%	0.13%
